# Effect of Transversely Isotropic Elasticity on Elastohydrodynamic Lubrication of Point Contacts

**DOI:** 10.3390/polym14173507

**Published:** 2022-08-26

**Authors:** Enzo Maier, Moritz Lengmüller, Thomas Lohner

**Affiliations:** Gear Research Center (FZG), Department of Mechanical Engineering, School of Engineering and Design, Technical University of Munich, Boltzmannstr. 15, D-85748 Garching near Munich, Germany

**Keywords:** short fiber reinforcement, polyamide (PA66), transversely isotropic elasticity, elastohydrodynamic lubrication (EHL), finite element method (FEM)

## Abstract

Fiber-reinforced materials or 3D printed parts feature transversely isotropic elasticity. Although its influence on pressures, shapes, and sizes has been studied extensively for dry contacts, the transferability to lubricated contacts is fragmented. This numerical study investigates how the content and orientation of short fibers in fiber-reinforced polymers (FRP) affect elastohydrodynamic lubrication (EHL) of point contacts. Material properties are modeled with Tandon-Weng homogenization. For EHL modeling, a fully-coupled approach based on finite element discretization is used. Results on hydrodynamic pressure and film thickness as well as material stress distribution are analyzed and compared to common approximations using the effective contact moduli. It is shown that the combination of fiber content and orientation defines the effective contact stiffness that determines the contact shape, size, and film thickness. Furthermore, the contact regime can change if a contact-specific stiffness threshold is reached.

## 1. Introduction

Machine elements made from plain technical polymers allow for highly efficient operation under lubricated conditions due to low stiffness and contact pressures. On the contrary, low strength and wear resistance constrain the application to low loads. Short fibers and fiber laminates are used to specifically reinforce technical polymers, and thus, increase power density, making them an indispensable part of many lightweight structures. For machine elements, short fiber reinforced polymers (FRP) can feature efficient tribological contacts with increased load-carrying capacity and cost-efficient injection-molding manufacturing. For example, experimental investigations by Kurokawa et al. [1] and Senthilvelan and Gananamoorthy [2,3] show lower wear rates, less thermal expansion, and an increased load-carrying capacity for fiber-reinforced spur gears, while unreinforced variants dampen meshing noise more effectively. While injection-molding has been identified as the ideal fabrication method for short FRP [4], alternatives are extrusion and additive manufacturing [5], both used in smaller batch sizes [6].

**Figure 1:**
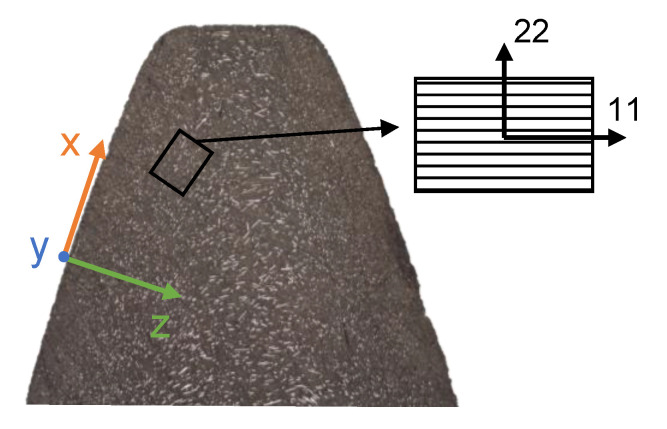
Cross-sectional microscopy of the short glass fibers orientation in an injection molded gear tooth (m_n_ = 3 mm) with schematic coordinate systems.

The fiber content, its distribution, and its orientation in machine elements are essential design criteria that link structural engineering, the tribological system, and manufacturing. Figure 1 shows an exemplary cross-section of an injection-molded polyamide gear tooth with short glass fiber reinforcement. While very few fibers are found in the proximity of the gear flanks, the content increases homogeneously in depth-direction (z). The fiber orientation (11) changes from out-of-the plane (y) to a random orientation mostly in the xz-plane. Fiber orientation and distribution are determined by the pressure and shear-driven flow of the compound polymer melt through the cavities of the tool system during the injection molding and the subsequent cool-down. This result in potential process-induced non-isotropic gear tooth stiffness (and thermal conductivity) and, thus, affect the tribological and structural behavior. Fiber distribution and orientations may result in a non-homogenous contact pattern that can cause local over-load.

To approximate tribological properties such as film thickness, pressures, and subsurface stresses in the engineering of parts with non-isotropic stiffness, analytical formulas are commonly used that were derived for isotropic or unidirectional stiffness orientations. The mathematical models to describe pressure and stress distribution, as well as the shape of dry or static contacts, were first derived in the 1950s. After the fundamental works of Hertz in 1882 [7], Willis [8] extended the Hertzian contact theory to generally anisotropic half-spaces based on the works of Green and Zerna [9]. For contacts with transversely isotropic elasticity, Turner [10] proposes an equivalent problem with an elastic transversely isotropic half-space and a rigid body. Transverse isotropy features a favored direction (11) which is the axis of symmetry (e.g., fiber direction) and a perpendicular plane with different isotropic material properties. Swanson [11] reduces Turner’s effective modulus to that for isotropic materials. To approximate the pressure and contact area of non-isotropic materials by the Hertzian theory, Yang and Sun [12] and Tan and Sun [13] propose to replace the isotropic modulus with the modulus of elasticity in the loading direction. For typical carbon fiber laminates, this can lead to deviations of up to 22% compared to Swanson’s approach [11].

The effective contact stiffness of non-isotropic materials determines contact shape and deformation. In elastohydrodynamically lubricated (EHL) contacts, this affects the hydrodynamics, and thus, the general contact behavior and contact regime. Only very few publications are found on EHL contacts with transversely isotropic materials. Chen et al. [14] use a finite element (FE) approach to investigate the influence of sliding speeds and loads in EHL contacts with transversely isotropic biological materials. Wang and Zhang [15] develop an efficient numerical approach that couples the solution of contour integrals as described for dry contacts with the Reynolds equation for hydrodynamics. Based on their film thickness analyses, they derive an effective elastic modulus for transversely isotropic materials. Zhao et al. [15] present a generalized and efficient modeling approach for soft EHL contacts with heterogenous bodies such as coated surfaces or FRPs that allows coupling to complex contact geometries, such as gears. Similarly, Paulson and Sadeghi [16] use an efficient FE modeling approach to demonstrate how randomly-oriented anisotropic crystals affect the stiffness and EHL contact behavior. To the author’s knowledge, a systematic analysis of EHL contacts with fiber-reinforced polymers and arbitrary orientation has not been performed despite growing interest in these materials.

This numerical study investigates how a preferred stiffness orientation of short fiber reinforced technical polymers affects the elastohydrodynamic lubrication of ball-on-flat rolling contacts in steady-state conditions. The focus lies on the effects of a variation of the degree of anisotropy (fiber content), its orientation, and orientation distribution on contact shape and size, film thickness, and stress distribution. This is significant to identify optimal fiber orientations for the engineering of efficient machine elements with non-isotropic materials and to improve manufacturing processes accordingly. A method is presented to determine minimum film thickness numerically, which is later compared to the Hamrock-Dowson approximation formulas. An exemplary distribution of fiber content and orientation derived from a tribometer sample links the conclusions to real parts.

## 2. Methods

This study considers an FE approach to calculate the isothermal EHL contact considering FRPs in a ball-on-flat configuration. Section 2.1 introduces the contact configuration, Section 2.2 and Section 2.3 the materials and lubricants as well as the reference operating condition. Section 2.4 presents the EHL model which consists of the governing equations for the lubricant hydrodynamics, the solid mechanics, and the load balance as well as the FE-discretized domains of the respective equations.

### 2.1. Contact Configuration

**Figure 2:**
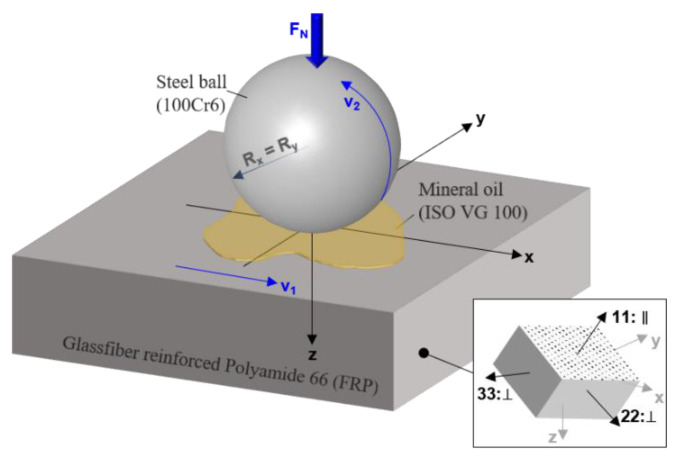
Schematic representation of the investigated contact configuration including the global (xyz)-coordinate system and the local (123)-coordinate system of the FRP.

Figure 2 visualizes the investigated contact configuration schematically, including the global and local coordinate systems used throughout this study. The steel ball (*R_x_ = R_y_* = 20 mm) is pressed onto the FRP flat space by a normal force *F_N_*. Both surfaces move in the same direction with ${\vec{v}}_{1}={\vec{v}}_{2}$ (*SRR* = 0%). Surfaces are assumed to be ideally smooth and are thermally stable at bulk temperature $\vartheta_{B}$. The contact is fully-flooded with mineral oil (ISO VG 100). The origin of the global Cartesian (xyz-)coordinate system lies on the flat in the center of the contact. The *x*-axis points in the direction of the mean entrainment speed ${\vec{v}}_{m}$ and the *z*-axis is parallel to the axis of loading and points into the FRP flat. The FRPs local (123-)coordinate system is orthonormal with the (11)-axis pointing in the fiber direction.

### 2.2. Materials

The steel ball is considered as 100Cr6 (1.3505). The flat is an FRP with a polyamide 66 (PA66) matrix and short glass fibers (E-glass) with variable weight content ϕ. Table 1 shows the materials’ mechanical properties. The FRP compound is heterogeneous because of the different mechanical properties of matrix and fiber. Note, material properties of PA66 may change significantly but are assumed constant, neglecting temperature- moisture- or age-dependency. As the direct modeling of both phases is inefficient in large geometries, homogenization approaches are used. Therein, the material properties are either derived from representative volume elements or theoretical models [17]. The resulting homogenized stiffness tensor is then aligned with pre-determined orientation tensors. The orientation tensors can be derived experimentally, e.g., from tomography or microscopy, see Figure 1, or numerically from melt flow simulations [18,19]. In this study, the compound stiffness is assumed to be transversally isotropic and elastic, which is appropriate for many engineering applications [20]. As the injection-molded fibers are typically shorter than 1 mm, the Tandon-Weng model [21] can be used to accurately calculate the five elastic constants [20] of the transversely isotropic stiffness tensor: the elastic modulus along the axis of symmetry E_11_, the in-plane elastic modulus E_22_, the shear modulus parallel to the axis of symmetry G_12_, the in-plane shear modulus G_23_, and the Poisson ratio ν_12_. The aspect ratio of the fibers can vary according to the injection-molding process and is assumed to be *a_f_* = *l_f_/d_f_* = 100. The presentation of the lengthy equations of the Tandon-Weng model can be found in [20,21] and likewise. Table 2 shows the calculated elastic constants for an increasing fiber content ϕ = {0,10,20,30} wt.%. Additionally, the degree of anisotropy A^U^ as defined by Ranganathan and Ostoja-Starzewski [22] is shown, which is null for the case of isotropy.

### 2.3. Lubricant

The contact is lubricated by the mineral oil MIN100, which was also used in other publications of the authors, e.g., [26,27,28]. The pressure- and temperature-dependence of viscosity $\eta \left(p,\;\vartheta_{B}\right)$ was modeled using the Roelands model [29], as shown in Equations (1) and (2) with the reference viscosity $\eta \left(\vartheta_{B}\right)$ obtained by the Vogel-Fulcher-Tamann (VFT) Equation (3), determined at the bulk temperature$\;\vartheta_{B}$. Based on the findings of [26,27,30], the shear stresses were estimated to be small and the lubricant rheology was assumed Newtonian, see Section 3. The pressure- and temperature-dependence of density $\rho \left(p,\;\vartheta_{B}\right)$ was modeled using Bode’s model [31] in Equation (4). The pressure-viscosity coefficient at bulk temperature is $\alpha_{p}\left(\vartheta_{B}\right)=27.7$ GPa^−1^. All lubricant properties and model coefficients are found in [27,28].

(1) $$\eta \left(p,\vartheta_{B}\right)=\eta \left(\vartheta_{B}\right)e^{\left(\ln\left(\eta \left(\vartheta_{B}\right)\right)+9.67\right)\left({\left(1+\frac{p}{p_{r}}\right)}^{Z}-1\right)}$$

(2) $$Z=p_{r}\alpha_{p}/\left(\ln\left(\eta \left(\vartheta_{B}\right)\right)+9.67\right)$$

(3) $$\eta \left(\vartheta_{B}\right)=A_{\eta }\exp\left(B_{\eta }/\left(\vartheta_{B}-C_{\eta }\right)\right)$$

(4) $$\rho \left(p,\vartheta_{B}\;\right)=\frac{\rho_{s}\cdot \left(1-\alpha_{s}\cdot \left(\vartheta_{B}+273.15\right)\right)}{1-D_{\rho 0}\cdot ln\left(\frac{D_{\rho 1}+D_{\rho 2}\cdot \left(\vartheta_{B}+273.15\right)+p}{D_{\rho 1}+D_{\rho 2}\cdot T}\right)}$$

### 2.4. Governing Equations

Reynolds equation describes the contact’s lubricant flow with unidirectional entrainment in gap length direction *x*. The isothermal version for point contacts with Newtonian lubricant rheology reads [32]:

(5) $$\nabla \cdot \left(\frac{\rho h^{3}}{12v_{m}\eta }\nabla p\right)=\frac{\partial \left(\rho h\right)}{\partial x}+\xi p^{-}$$

The factor ξ = 10^6^ penalizes negative pressures $p^{-}$ to prevent cavitation. Equation (6) describes the lubricant film geometry.

(6) $$h\left(x,y\right)=h_{0}+\frac{x^{2}+y^{2}}{2R}+\delta_{1}\left(x,y\right)+\delta_{2}\left(x,y\right)$$

Therein, a paraboloid describes the shape of the undeformed solid body surfaces with its curvature determined by reduced radius *R*. The rigid body offset *h*_0_ is adjusted such that the external force balances the hydrodynamic pressure:

(7) $$\int^{\;}p\left(x,y\right)\;d\Omega_{p}=F_{N}$$

The elastic deformations of the surfaces $\delta_{1,2}$ are calculated separately to evaluate the respective stress states and are obtained by solving Hooke’s elasticity Equation (8):

(8) $$\nabla \cdot {\underline{\sigma }}_{i}=0\;{\underline{\sigma }}_{i}=C_{i}^{\mathrm{xyz}}\cdot \underline{\varepsilon }\left({\vec{u}}_{i}\right)=-p\cdot \underline{1}+{\underline{\sigma }}_{dev}\;{\vec{u}}_{i}=\left(\begin{array}{c} u_{x,i}\\ u_{y,i}\\ \delta_{i} \end{array}\right),\;i=\{\begin{array}{c} 1:\;\mathrm{flat}\\ 2:\;\mathrm{ball} \end{array}$$

The FRPs’ transversally isotropic compliance tensors ***C_1_^−1^*** in Voigt notation and fiber coordinate system reads:

(9) $$\left(\begin{array}{c} \begin{array}{c} \varepsilon_{11}\\ \varepsilon_{22}\\ \varepsilon_{33} \end{array}\\ \begin{array}{c} \varepsilon_{23}\\ \varepsilon_{13}\\ \varepsilon_{12} \end{array} \end{array}\right)=\underset{\underset{{C_{1}^{123}}^{-1}}{︸}}{\left(\begin{array}{cc} \begin{array}{ccc} 1/E_{11} & -\nu_{21}/E_{11} & -\nu_{21}/E_{11}\\ -\nu_{12}/E_{22} & 1/E_{22} & -\nu_{23}/E_{22}\\ -\nu_{12}/E_{22} & -\nu_{23}/E_{22} & 1/E_{22} \end{array} & 0\\ 0 & \begin{array}{ccc} 1/G_{23} & 0 & 0\\ 0 & 1/G_{12} & 0\\ 0 & 0 & 1/G_{12} \end{array} \end{array}\right)}\left(\begin{array}{c} \begin{array}{c} \sigma_{11}\\ \sigma_{22}\\ \sigma_{33} \end{array}\\ \begin{array}{c} \sigma_{23}\\ \sigma_{13}\\ \sigma_{12} \end{array} \end{array}\right)$$

Note that $\nu_{21}/E_{11}=\nu_{12}/E_{22}$.

To rotate the fiber coordinate system in the global coordinate system, Euler transformation (z-x’-z’’ order) is applied with the rotation matrices ***R***:

(10) $$C_{1}^{\mathrm{xyz}}=R^{z}{}^{\prime \prime }\left(\gamma \right)R^{x^{\prime }}\left(\beta \right)R^{z}\left(\alpha \right)\cdot C_{1}^{123}\cdot R^{z}\left(\alpha \right)R^{x^{\prime }}\left(\beta \right)R^{z}{}^{\prime \prime }\left(\gamma \right)$$

The steel ball is modeled as isotropic and elastic such that the stiffness tensor ***C_2_*** in Voigt notation reads:

(11) $$\left(\begin{array}{c} \begin{array}{c} \sigma_{xx}\\ \sigma_{yy}\\ \sigma_{zz} \end{array}\\ \begin{array}{c} \sigma_{yz}\\ \sigma_{xz}\\ \sigma_{xy} \end{array} \end{array}\right)=\frac{E}{\left(1+\nu \right)\left(1-2\nu \right)}\left(\begin{array}{cc} \begin{array}{ccc} 1-\nu & \nu & \nu \\ \nu & 1-\nu & \nu \\ \nu & \nu & 1-\nu \end{array} & 0\\ 0 & \begin{array}{ccc} \frac{1-2\nu }{2} & 0 & 0\\ 0 & \frac{1-2\nu }{2} & 0\\ 0 & 0 & \frac{1-2\nu }{2} \end{array} \end{array}\right)\left(\begin{array}{c} \begin{array}{c} \varepsilon_{xx}\\ \varepsilon_{yy}\\ \varepsilon_{zz} \end{array}\\ \begin{array}{c} \varepsilon_{yz}\\ \varepsilon_{xz}\\ \varepsilon_{xy} \end{array} \end{array}\right)$$

### 2.5. Computational Domain

**Figure 3:**
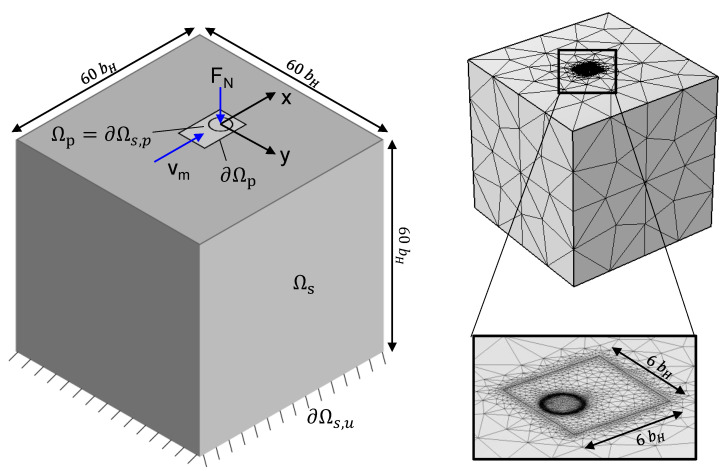
Domain (left) and mesh (right) of the FE isothermal EHL model.

Figure 3 shows the geometric domains of the governing equations and the finite element mesh. Therein, the hydrodynamic pressure distribution is solved for in $\Omega_{p}$, the contact body deformations in $\Omega_{s}$, and the rigid body offset in $\Omega_{p}$. Any other quantities, such as stresses and film thickness profiles are derived from these. Zero-pressure boundary conditions apply to $\partial \Omega_{p}$ and zero-displacement boundary conditions apply to $\partial \Omega_{s,u}$. The domain of the Reynolds equation coincides with the traction boundary condition on the solids $\partial \Omega_{s,p}=\Omega_{p}$. The solid edges are sufficiently far away from the contact center (approx. 30 times the contact radius) such that the zero-traction boundary condition on the remaining solid boundaries does not affect the contact deformation.

Equations (5), (7), and (8) are discretized separately using the finite element method and coupled through equation (6). Streamline-Upwind Petrov-Galerkin [33] and Galerkin-Least-squares stabilizations [34] are applied to the Reynolds Equation (5). COMSOL Multiphysics solves the fully-coupled discretized system of equations in a Newton-Raphson scheme with MUMPS linear solver [35].

### 2.6. Operating Condition and Parametric Variations

**Figure 4:**
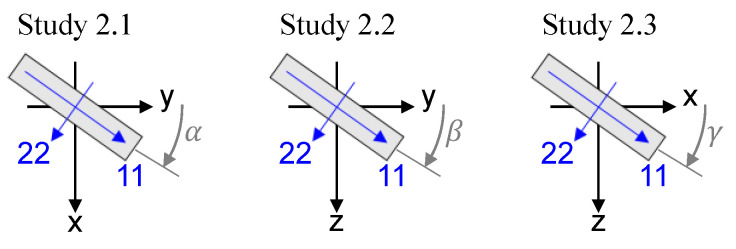
Visualization of the fiber orientations in study 2.

The considered operating condition and parameter variations are shown in Table 3. The reference configuration features a compromise for load and speed to compare unreinforced and FRP materials. The reference fiber orientation (11) is parallel to the y-direction, see Figure 2. In study 1, the fiber content is reduced to the unreinforced isotropic case. Study 2 investigates the rotation of the reference configuration: in the xy-plane around the global *z*-axis (study 2.1), in the yz-plane around the global *x*-axis (study 2.2), and the rotation of the fiber direction (11) from parallel to the *x*-axis to parallel to the *z*-axis in the xz-plane (study 2.3). Figure 4 visualizes the fiber orientations of study 2 for clarity. Lastly, study 3 investigates a distribution of orientations and fibers that were derived from an injection-molded tribometer specimen of a twin-disk tribometer [30], similar to Figure 1.

### 2.7. Analytical Approximations

For reference purposes, the minimum film thickness is calculated by Hamrock-Dowson equations [36] for piezoviscous-elastic (PE) and isoviscous-elastic (IE) contact regimes of point contacts.

(12) $$\mathrm{IE}\;\mathrm{regime}:\;H_{\min}=\frac{h_{\min}}{R}{\left(\frac{W}{U}\right)}^{2}=8.70\frac{W^{1.79}}{U^{1.34}}\left(1-0.85e^{-0.31k}\right)$$

(13) $$\mathrm{PE}\;\mathrm{regime}:\;H_{\min}=\frac{h_{\min}}{R}{\left(\frac{W}{U}\right)}^{2}=3.63\frac{G^{0.49}U^{0.68}}{W^{0.073}}\left(1-e^{-0.68k}\right)$$

(14) $$k=1.03{\left(\frac{R_{x}}{R_{y}}\right)}^{0.64},\;U=\frac{\eta \left(\vartheta_{B}\right)v_{m}}{{RE}^{\prime }},\;G=\alpha_{p}\left(\vartheta_{B}\right)E^{\prime },\;W=\frac{F_{N}}{R^{2}E^{\prime }}$$

The dimensionless parameters *U*, *G*, and *W* relate to the viscosity $\eta \left(\vartheta_{B}\right)$, the pressure-viscosity coefficient $\alpha_{p}\left(\vartheta_{B}\right)$ and the operating condition, presented in Section 2.3. The reduced elastic modulus *E′* summarizes the elastic properties of the steel ball and the FRP flat. In this study, it is defined as follows with *i* as FRP and *j* as 100Cr6:

(15) $$E^{\prime }=\frac{2}{\hat{E_{l}}+\frac{1-\nu_{j}^{2}}{E_{j}}}$$

(16) $$\hat{E_{l}}=\{\begin{array}{c} \begin{array}{c} E_{T}\\ E_{W+Z} \end{array}\\ \begin{array}{c} E_{E11}=\frac{1-\nu_{12}^{2}}{E_{11}}\\ E_{E33}=\frac{1-\nu_{23}^{2}}{E_{33}} \end{array} \end{array}$$

The effective elastic moduli *E_T_* and *E_W+Z_* are functions of the transversely isotropic elastic constants presented in Table 2 and are defined by Turner [10] and Wang and Zhang [15], respectively. *E_E_*_11_ and *E_E_*_33_ originate from Yang and Sun [12] and Tan and Sun [13]. Another set of dimensionless parameters is the elasticity and viscosity parameters *g_E_* and *g_V_* defined by Johnson [37]:

(17) gE=W83U2, gV=GW3U2

The solids’ stressing is determined by von Mises stresses, which are defined in terms of principal stresses $\sigma_{1/2/3}$ as follows:

(18) $$\sigma_{mises}=\sqrt{\frac{{\left(\sigma_{1}-\sigma_{2}\right)}^{2}+{\left(\sigma_{2}-\sigma_{3}\right)}^{2}+{\left(\sigma_{3}-\sigma_{1}\right)}^{2}}{2}}\;$$

## 3. Results

This section presents the studies’ results and discusses pressure distribution, film thickness profiles, deformation, and subsurface stresses.

### 3.1. Increase in Fiber Content (Study 1)

**Figure 5:**
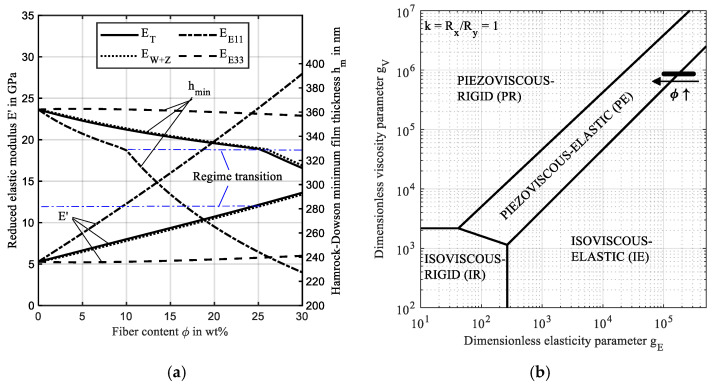
(**a**) Reduced elastic modulus E’ and minimum film thickness for the FRP effective elastic modulus models by Turner [10] ($E_{T}$), Wang and Zhang [15] ($E_{W+Z}$), Yang and Sun [12] ($E_{E11}$) and Tan and Sun [13] ($E_{E33}$) for variation of fiber content; (**b**) contact regime characterization of the investigated ball-on-flat contact in Johnson’s map [37], redrawn from [38].

Figure 5a compares the moduli and the resulting minimum film thickness for an increasing fiber content based on the analytical approximations, according to Section 2.7. As expected, the reduced stiffness *E′* increases in all models, and more than fivefold, if the effective stiffness $\hat{E}$ is approximated by the stiffness in the fiber direction $E_{E11}$. The differences between $E_{T}$ and $E_{W+Z}$ are less than 1.6%. The corresponding minimum film thickness decreases according to Equations (12) and (13) and shows a maximum difference of 130 nm at the highest fiber content between $E_{E11}$ and $E_{E33}$, as a result of the regime change. A fiber content ϕ<15wt.% causes a reduced stiffness in the isotropic plane according to the Tandon-Weng model [21], which can be justified by the notch effect of fiber inclusions in the matrix. As a result, the film thickness for *E_E_*_33_ increases slightly before it decreases for ϕ≥15 wt.%. Figure 5b characterizes the investigated contacts in Johnson’s map [37]. The point farthest to the right corresponds to the isotropic case, whereas the farthest left point corresponds to the highest fiber content aligned in contact normal direction.

The contact regime transitions from IE to PE for high fiber content and stiffness. This can be seen in Figure 5a where the transition occurs at 10 wt.%, 25 wt.%, and 26 wt.% for the *E_E_*_11_, *E_T_*_,_ and *E_W+Z_* models, respectively. Thus, the transition occurs at a reduced elastic modulus *E′* of approx. 12.2 GPa, which corresponds to a minimum film thickness of approx. 330 nm or a Hertzian pressure of approx. 102 MPa for the given operating condition. Note, that the transition point is not related to physical phenomena in the EHL contact and rather a transition region. The predicted reduced elastic modulus *E*’ and the film thicknesses vary significantly between the investigated analytical approximations. Consequently, the commonly used dimensionless result representation with Hertzian pressures *p_H_* and contact radius *a* is omitted in the following.

**Figure 6:**
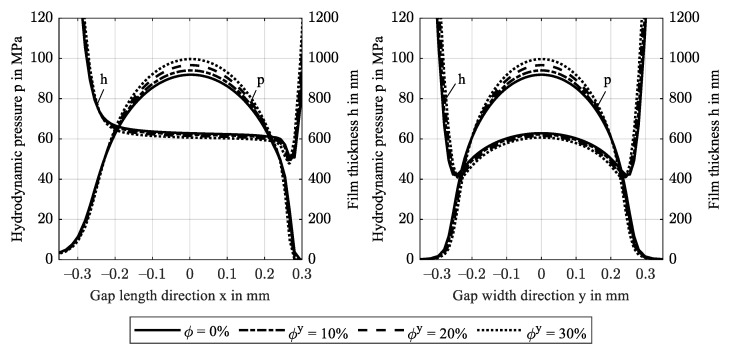
Hydrodynamic pressure *p* and film thickness profiles *h* along the gap length x at y = 0 (**left**) and along the gap width y at x = 0 (**right**) for increasing fiber content in the y-direction $\phi^{y}$.

**Figure 7:**
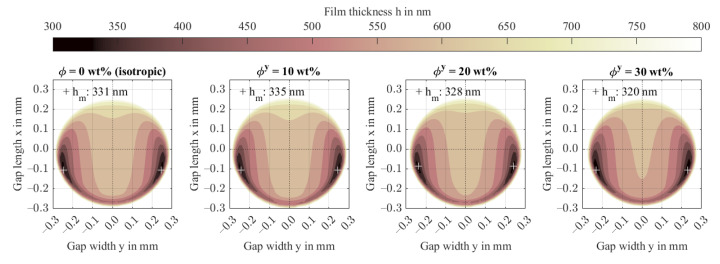
Film thickness contours including minima for increasing fiber content in the y-direction $\phi^{y}$.

Based on the EHL model, Figure 6 shows the hydrodynamic pressure distribution *p* and film thickness profiles *h* along the central gap length x (left) and along the central width y (right) for increasing fiber content in gap width direction y. The general pressure profile is typical for isothermal EHL contacts in the IE contact regime: In the inlet region, the moving surfaces drag lubricant into the contact region to form a lubricating film while pressure increases from the ambient atmosphere. In the contact region, the pressure profile resembles the elliptical shape known from dry contacts with its maximum pressure in the contact center. Due to mechanical compliance, the external load is distributed over a large contact area such that maximum pressures remain below 100 MPa. In the outlet region, pressure drops to the ambient atmosphere. The film thickness profile forms a parallel gap in the central contact region. Superposition of the shear flow from moving surfaces and pressure-driven flow forces a film constriction at the end of the contact region and a related sudden pressure drop. As a result of the low pressure-viscosity increase, no second pressure maximum occurs. In EHL point contacts, the entrainment flow in the x-direction diverges such that the minimum film thickness is found at an offset from the *x*-axis in the contact center, see Figure 6 (right) and Figure 7. The constant reference fiber orientation parallel to the *y*-axis causes pressure distribution and film profile to be symmetrical with the xz-plane. The results show that an increase in fiber content $\phi^{y}$ leads to higher pressures and lower film thicknesses. This is due to an increase in the effective stiffness of the solid and agrees with Figure 5.

Figure 7 shows film thickness contours. The minimum film thickness decreases to 320 nm for $\phi^{y}$ = 30 wt.%, which corresponds to the predicted value in Figure 5. At low fiber content, the minimum film thicknesses deviate from the predicted values which is attributed to the Tandon-Weng homogenization approach. Therein, the calculated transverse stiffness causes lower minimum film thicknesses compared to when the stiffness models of Equation (13) are used. The off-axis position and the contact size are barely affected by the increasing anisotropy.

**Figure 8:**
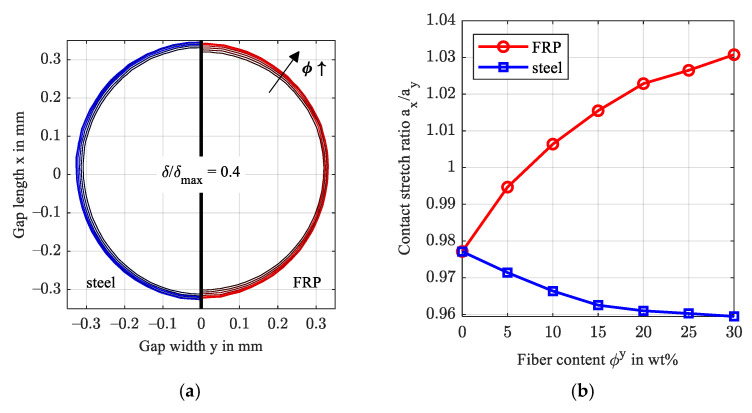
Normalized contact deformation of the steel and FRP surface for increasing fiber content in the y-direction (**a**) and contact stretch ratio (**b**).

Figure 8 evaluates the elliptic shape of the normalized contact deformation of steel and FRP just outside the contact area as a ratio of the widest stretch in x- and y-directions a_x_/a_y_. Therein, the deformation was normalized by maximum deformation and is plotted at a ratio of 40% ($\delta /\delta_{\max,i}=0.4$). Beginning at a ratio just below 0.98 attributed to the asymmetrical pressure distribution in gap length direction x, the FRP deformation stretches in the x-direction with rising fiber content in the y-direction. Contrarily, the normalized deformation of the steel surface stretches in the x-direction at a much smaller magnitude. This ellipticity agrees with the findings for arbitrarily oriented transversely isotropic half-spaces, e.g., Fabrikant [39] and Swanson [11]. Along with the deformation, the directional dependency affects the stresses in the FRP.

**Figure 9:**
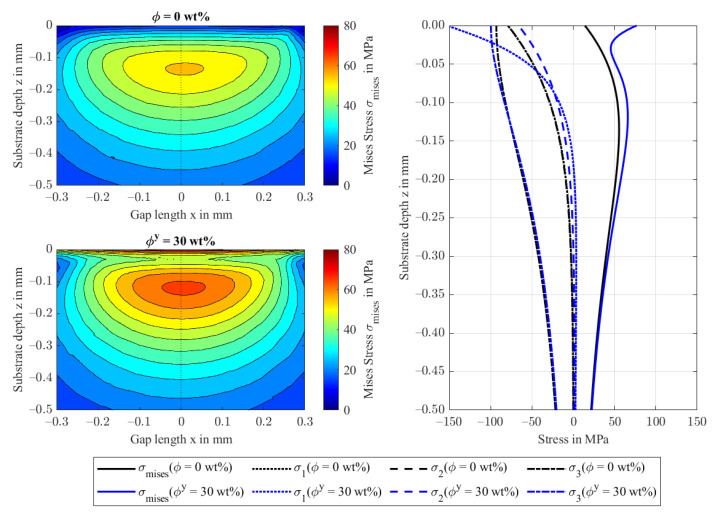
von Mises stresses in the xz-plane at y = 0 for $\phi^{y}$ = 0 and 30 wt.% in the y-direction (**left**) and the respective principal stresses of the FRP substrate in depth direction z (**right**).

Figure 9 visualizes the von Mises stresses $\sigma_{\mathrm{mises}}$ in the xz-plane for $\phi^{y}$ = 0 wt.% and 30 wt.% and the principal stresses $\sigma_{1/2/3}$, respectively. The maximum von Mises stress is higher in the FRP ($\phi^{y}=30$ wt.%) because of the increased stiffness and the resulting smaller contact area. As stresses are strongly multiaxial in the proximity of the surface, neither the shape modification hypothesis (von Mises stresses) nor the maximum principal stress hypothesis applies to the contacts, and other failure criteria are needed for FRPs [20]. Neglecting the von Mises stresses in the proximity of the surface, a maximum of $\sigma_{mises}=66\;\mathrm{MPa}$ is reached $\phi^{z}=30\;$wt.% at a depth of 0.120 mm. The isotropic case with ϕ=0 wt.% reaches its maximum at 55 MPa at a depth of 0.135 mm. Decomposition of the Mises stresses through Equation (11) explains the stress maxima by the large difference of principal stresses. In the isotropic case, the first and the second principal stresses $\sigma_{1/2}$ are identical along the depth direction and the third principal stress $\sigma_{3}$ corresponds to the applied hydrodynamic pressure boundary condition. In the transversely isotropic case, the third principal stress $\sigma_{3}$ behaves similarly, while the first and second principal stresses $\sigma_{1/2}$ show large differences as a result of the different stiffnesses (and Poisson ratios) in fiber direction 11 and in the orthogonal direction 22.

### 3.2. Fiber Rotation from Gap Width to Length Direction (Study 2.1)

**Figure 10:**
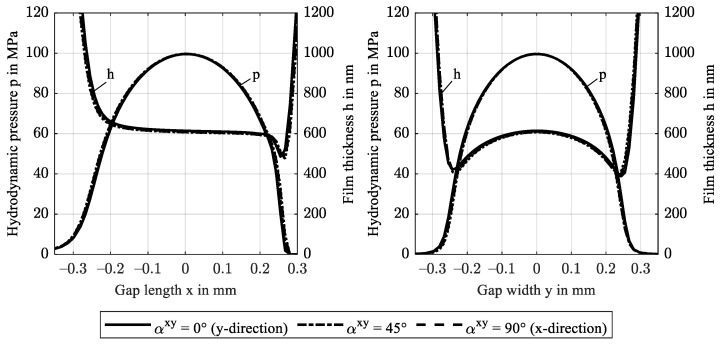
Hydrodynamic pressure *p* and film thickness profiles *h* along the gap length x at y = 0 (**left**) and along the gap width y at x = 0 (**right**) for a rotation $\alpha^{xy}$ of fiber orientation in the xy-plane.

This section investigates how various fiber orientations affect EHL contacts, see Table 3. Figure 10 shows the hydrodynamic pressure distribution *p* and film thickness profile *h* in gap length direction x and gap width direction y. It confirms that a rotation around any axis in the isotropic plane of a transversely isotropic FRP barely affects the hydrodynamic pressure distribution and the film thickness. This is a result of the isotropic stiffness in the (22)-direction.

**Figure 11:**
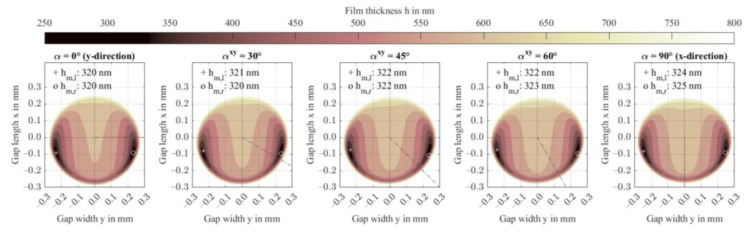
Film thickness contours including minima for variation of the fiber orientation $\alpha^{xy}$ in the xy-plane, as indicated by the dashed line.

Figure 11 shows the film thickness profiles including the minima on each side of the *x*-axis. Fiber orientation in entrainment direction x, or fractions thereof, can lead to slightly higher minimum film thickness than orientation in gap width direction y. This is explained by the symmetry of the pressure profile in gap width direction y and the asymmetry of the profile along the entrainment direction x, which determine the deformation profiles.

**Figure 12:**
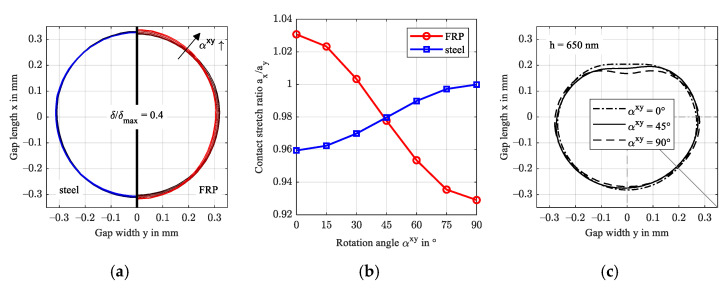
Normalized contact deformation of the steel and FRP surface for the increasing rotation angle $\alpha^{xy}$ in the xy-plane (**a**), contact stretch ratio (**b**), and isocontour at a film thickness level of 650 nm (**c**).

Figure 12 shows that the normalized FRP deformation profiles stretch perpendicular to the fiber direction (a) and at a similar magnitude (b). The steel profile stretches contrarily to FRP and the maximum deformations are approx. two orders of magnitude smaller. Figure 12c shows the isocontour at h = 650 nm for reference orientation ($\alpha^{xy}\;$= 0°), in x direction ($\alpha^{xy}$ = 90°) and at an intermediate angle $\alpha^{xy}$ = 45°. The latter deformation isocontour is asymmetrical with the central contact axis x (y = 0) due to the fiber orientation. As a result, the lubricant flow is slightly diverted which affects the magnitude and location of the minimum film thickness, see also Figure 11. Evaluation of continuity (mass flow) in the y-direction indicates a maximum change at $\alpha^{xy}$ = 45° (1.17 mg/s compared to 0.39 mg/s for the reference configuration). While the effect of the increased mass flow is as small as the rounding error in this study, a more pronounced effect is expected if both contact bodies feature (commonly oriented) strong anisotropic behavior.

**Figure 13:**
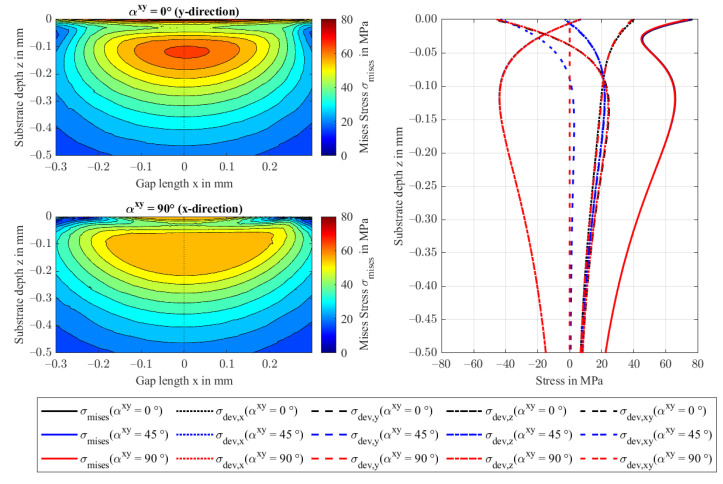
von Mises stresses in the xz-plane at y = 0 for $\alpha^{xy}$ = 0 and 90° (**left**) and the respective deviatoric stresses at the contact center of the FRP in substrate depth direction z (**right**).

The deviatoric stresses and von Mises stresses for rotations in the xy-plane are visualized in Figure 13. Upon a 90-degree rotation, the deviatoric x stress component transitions to become a deviatoric y stress component, and vice versa. At the 45° rotation, the deviatoric xy-component balances the weaker x and y components. The deviatoric z stress component and the mises stresses remain unchanged. The von Mises stress distributions (left) stretch in fiber direction and are lower in magnitude.

### 3.3. Fiber Rotation from Gap Width to Gap Normal Direction (Study 2.2)

**Figure 14:**
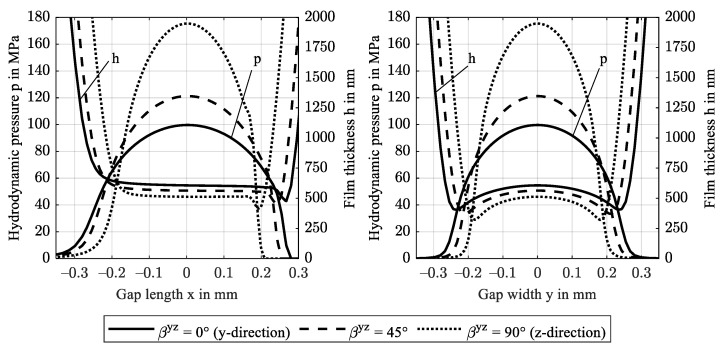
Hydrodynamic pressure p and film thickness h profiles along the gap length x at y = 0 (**left**) and along the gap width y at x = 0 (**right**) for rotation $\beta^{yz}$ of fiber orientation in the yz-plane.

**Figure 15:**
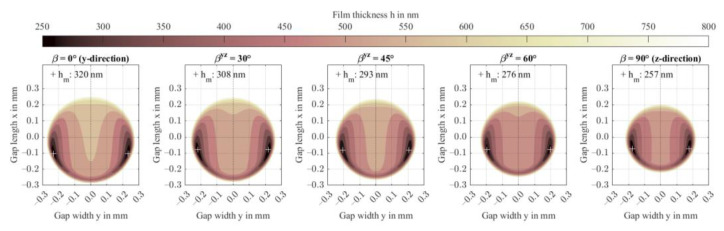
Film thickness contours including minima for variation of the rotation $\beta^{yz}$ of fiber orientation in the yz-plane.

If the material is assumed transversely isotropic and fibers are directed in contact normal direction z, the high stiffness leads to small contact areas and corresponding high pressures. Figure 14 shows the pressure distribution *p* and film thickness profiles *h* for an increasing rotation angle $\beta^{yz}$ from 0° (y-direction) to 90° (z-direction). At maximum, the hydrodynamic pressure distribution reaches 175 MPa in the contact center. The EHL contact then operates in the piezoviscous-elastic contact regime, as a second pressure maximum in front of the constriction occurs and the minimum film thickness is below 330 nm, see Section 3.1. Figure 15 shows the corresponding film thickness contours including the location of the respective minima. With fiber orientation in contact normal z-direction ($\beta^{yz}$ = 90°), minimum film thickness decreases to 257 nm. All profiles are symmetric to the central contact *x*-axis (y = 0).

**Figure 16:**
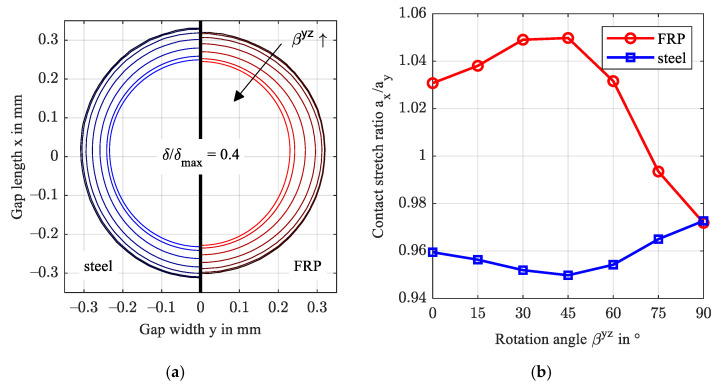
Normalized contact deformation of the steel and FRP surface for increasing rotation $\beta^{yz}$ of fiber orientation in the yz-plane (**a**) and contact stretch ratio (**b**).

**Figure 17:**
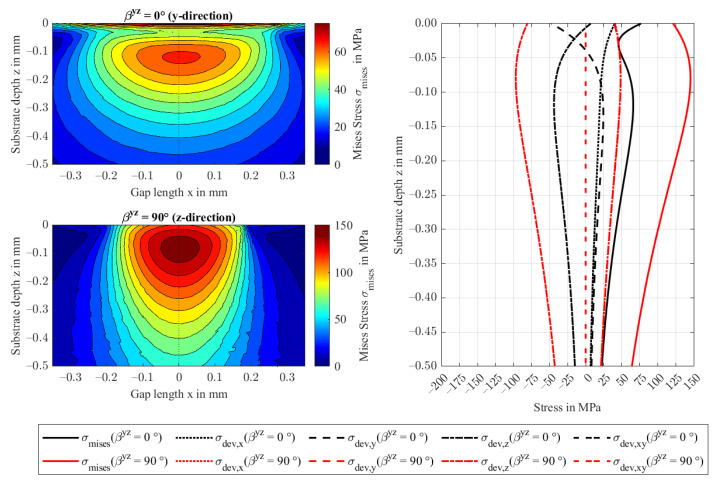
von Mises stresses in the yz-plane at y = 0 for $\beta^{yz}$ = 0 and 90° (**left**) and the respective principal stresses at the contact center of the FRP in substrate depth direction z (**right**).

Figure 16 presents the normalized deformation profiles. With increasing rotation angle $\beta^{yz}$, or orientation fractions in contact normal z-direction, the contact becomes stiffer. Consequently, the fiber orientation shows the same contact stretch ratio *a_x_/a_y_* as an isotropic contact and the contact shape is close to circular. Note, at $\beta^{yz}=45{}^{\circ}$, the contact stretch is the largest in this study and the material behaves most anisotropic amongst all performed studies. The von Mises stresses shown in Figure 17 confirm the findings shown in the previous sections. While the principal stresses transition to the isotropic case, see also Figure 9, the maximum von Mises stress reaches 146 MPa at $\beta^{yz}=90{}^{\circ}$ at a depth of 0.08 mm. The distribution stretches into the depth and narrows in the orthogonal directions.

**Figure A1:**
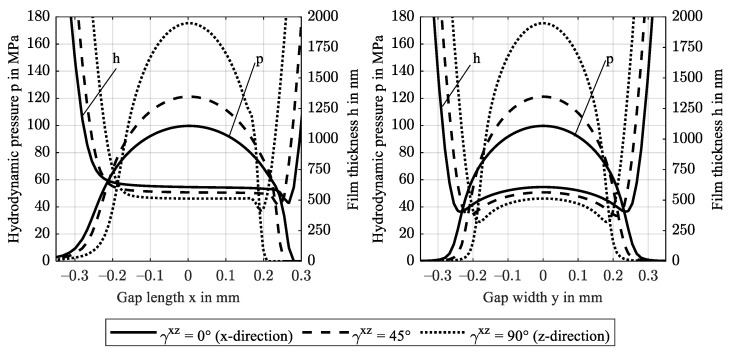
Hydrodynamic pressure *p* and film thickness profiles *h* along the gap length x at y = 0 (**left**) and along the gap width y at x = 0 (**right**) for rotation $\gamma^{xz}\;$ of fiber orientation from x- to z-direction.

**Figure A2:**
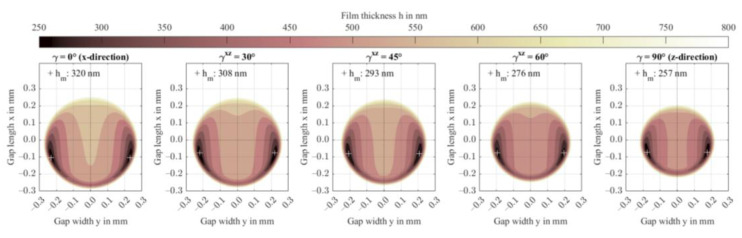
Film thickness contours including minima for variation of the fiber orientation $\gamma^{xz}$ in the xz-plane.

**Figure A3:**
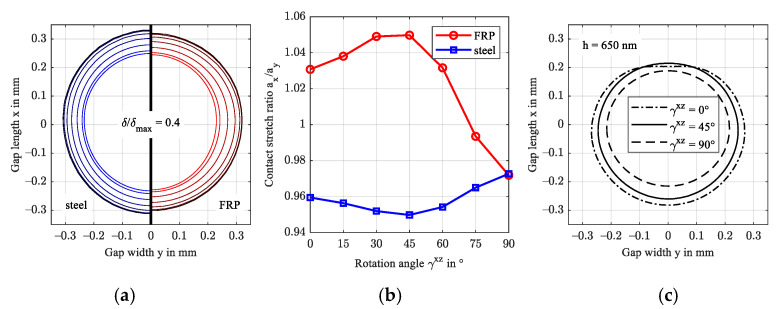
Normalized contact deformation of the steel and FRP surface for increasing rotation angle in the xy-plane (**a**) and contact stretch ratio (**b**) and isocontour of a film thickness level of 650 nm (**c**).

**Figure A4:**
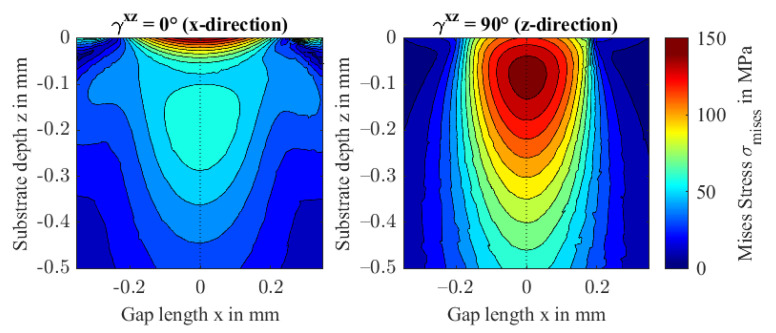
von Mises stresses in the xz-plane at y = 0 for $\gamma^{yz}$ = 0 and 90°.

The rotation $\gamma^{xz}$ of fiber direction from gap length direction x to contact normal direction z (study 2.3) confirms the above findings, showing no significant differences to study 2.2. Pressure distribution and film thickness profiles can be found in Figure A1 and Figure A2 along with the normalized deformation and von Mises stress distribution in Figure A3 and Figure A4 in the Appendix A.

### 3.4. Fiber Content and Orientation Distribution (Study 3)

**Figure 18:**
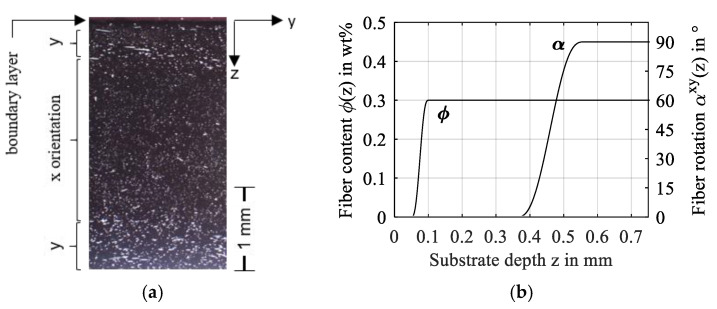
Cross-sectional microscopy of the fiber orientation in an injection-molded tribometer sample made of PA66 with 30 wt% glass fibers (**a**) and fiber content and orientation (**b**).

Injection-molded technical FRP parts can feature an amorphous boundary layer with little to no fibers and variation of fiber orientation in the depth direction. This results from the polymer melt’s pressure and shear flow through the injection molding cavities. Study 3 approximates the boundary layer thickness of an injection-molded tribometer sample at 0.1 mm with ϕ=0 wt%, before it ramps to ϕ=30 wt.%, see Figure 18. In the fiber-reinforced region, the orientation $\alpha^{xy}$ changes in depth direction z, which is modeled as a smoothed step function that rotates the fiber orientation from gap width direction y to entrainment direction x.

**Figure 19:**
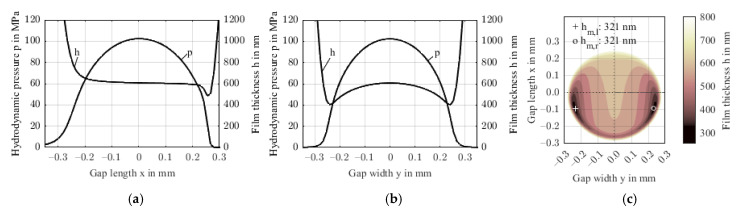
Pressure and film thickness profiles along the gap length x (**a**) and gap width y (**b**) and film thickness contour in the xy-plane including minimum (**c**) for distributed fiber content and orientation acc. to Figure 18.

**Figure 20:**
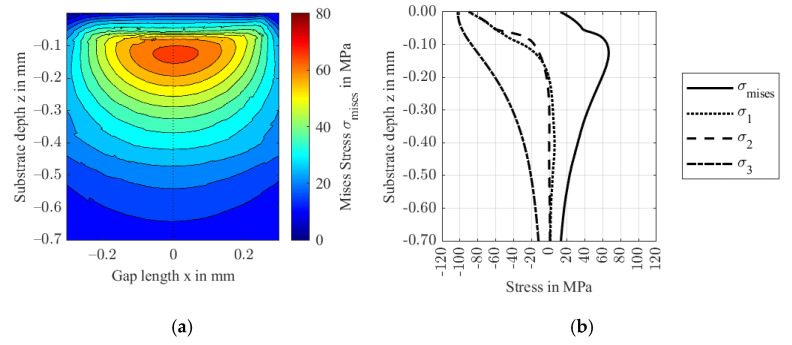
von Mises stresses in the xz-plane at y = 0 (**a**) and the respective principal stresses at the contact center of the FRP in substrate depth direction z (**b**).

As fiber content increases closely below the surface, the effect on the stiffness is small. The effective stiffness is also not significantly affected, because the fiber orientation rotates only around an axis in the isotropic plane. Hence, the results shown in Figure 19 compare to Study 2.1 (Figure 10 and Figure 11) with a minimum film thickness of 321 nm and a maximum hydrodynamic pressure of 102 MPa. Figure 20 shows the stress distribution in the FRP substrate material.

The stress distribution shows an almost isotropic behavior. Due to the unreinforced (isotropic) boundary layer, stresses in the proximity of the surface are relieved. The increase in fiber content leads to a small dent in the second principal stress profile $\sigma_{2}$ and consequently in the von Mises stress $\sigma_{mises}$. The latter’s maximum of 67 MPa is reached at a depth of 0.127 mm. The second rotation at a depth of 0.5 mm to an orientation in y-orientation does not affect the stress distribution significantly because of its distance to the contact zone.

### 3.5. Comparison to Analytical Formulas

**Figure 21:**
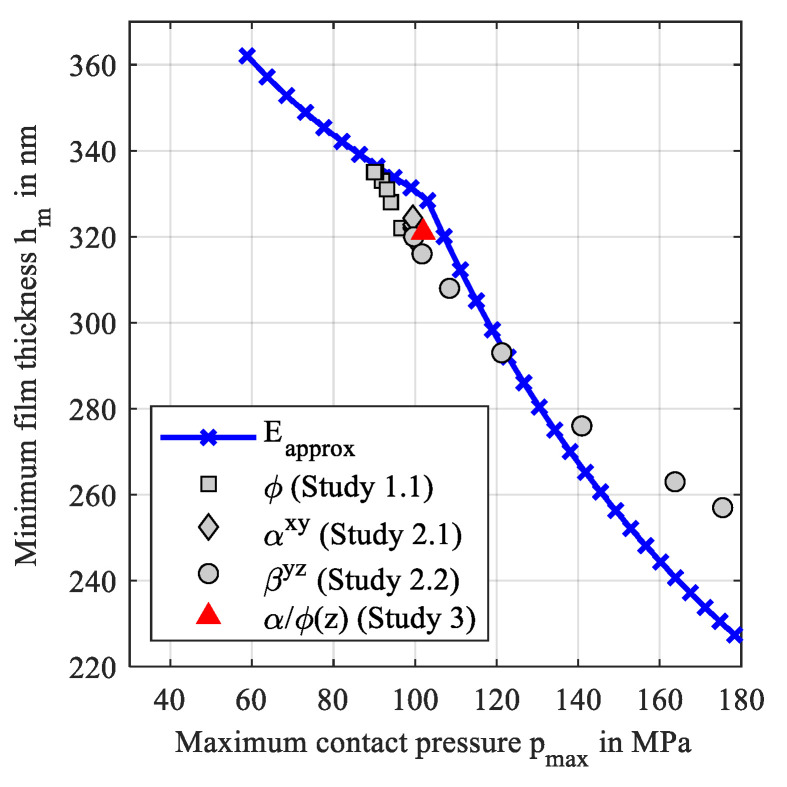
Estimated and calculated minimum film thicknesses over the maximum contact pressures.

To summarize the above findings, Figure 21 shows a comparison of the analytical approximations of the minimum film thickness according to Equations (12) and (13) and the numerically calculated film thicknesses of the studies 1.1, 2.1, 2.2, and 3, over the maximum contact pressure. The latter was calculated with Hertzian theory for the analytical approximations.

The analytical approximations show a dent which refers to the transition from IE to PE contact regime. The investigated orientations led to contact regimes close to the transition and resulted in lower minimum film thicknesses than the analytical approximations. In contrast, minimum film thicknesses are higher than approximations at high pressures.

## 4. Conclusions

The short fiber content and orientation affect the degree of non-isotropy, the effective contact stiffness, and the shape of the contact region of EHL contacts. Any stiffness increase leads to less surface normal deformation and thus lower film thickness, smaller contact size, higher pressure, and higher stresses. Based on this, the following can be concluded from the investigations in this study of transversely isotropic elasticity:

- The contact shape can affect the lubricant hydrodynamics. As a result, the contact regime may transition from isoviscous-elastic to piezoviscous-elastic at a threshold that depends on the operating condition.
- A fiber orientation parallel to the contact plane results in a stretch of the contact area and higher film thickness compared to an orientation perpendicular to the contact plane.
- The accuracy of analytical approximations of the minimum film thickness depends strongly on the effective contact stiffness, which depends on the homogenized fiber orientation and distribution.
- Subsurface stresses increase with fiber content. Fiber orientations in the contact plane can cause high von Mises stress in the proximity of the surface, while fiber orientations in the contact normal direction result in maximum von Mises stress at higher depths.
- The typical fiber orientation distribution after injection molding may effectively lead to isotropic-like contact behavior.

The presented results can be used to design and engineer fiber-reinforced and additively manufactured parts with predetermined contact patterns. The method and the findings can be also used in strength verification if combined with an appropriate failure criterion, as well as in the improvement of the manufacturing processes for tribologically loaded parts. Future research needs to focus on a critical review of the Tandon-Weng homogenization approach including an experimental validation and the application of the findings to machine elements such as gears.

## Figures and Tables

**Table 1:** Considered mechanical properties of steel ball and matrix and fiber phases FRP.

	Material	Elastic Modulus	Poisson Ratio	Density
Steel	100Cr6	210,000 MPa [23]	0.30 [23]	7800 kg/m^3^ [23]
Matrix	PA66	2350 MPa [24] *	0.35 [25]	1140 kg/m^3^ [6]
Fiber	E-Glass	72,500 MPa [20]	0.20 [20]	2550 kg/m^3^ [20]

* Interpolated for operating temperature.

**Table 2:** Elastic constants and degree of anisotropy for transversely isotropic FRP with varying fiber content, calculated with the Tandon-Weng model [21] and according to [22].

Fiber Content	E_11_ in MPa	E_22_ in MPa	G_12_ in MPa	G_23_ in MPa	$\nu_{12}$	A^U^
0 wt.%	2350	2350	870.4	870.4	0.35	0.000
10 wt.%	5576	2359	951.4	937.3	0.34	0.699
20 wt.%	9214	2507	1053.0	1020.4	0.33	1.672
30 wt.%	13,347	2724	1182.1	1126.7	0.32	2.614

**Table 3:** Operating condition and parameter variations of the reference and the parametric studies.

		Unit	Ref.	Study 1	Study 2.1	Study 2.2	Study 2.3	Study 3
Normal force	F_N_	N	15					
Mean speed	v_m_	m/s	1					
Bulk temperature	$\vartheta_{B}$	°C	40					
Fiber content	ϕ	wt.%	30	0…30	30			ϕ(z)
Fiber orientation *	$\alpha^{xy}$	°	0	0	0…−90	0	−90	$\alpha^{xy}\left(z\right)$
	$\beta^{yz}$	°	0	0	0	90	90	0
	$\gamma^{xz}$	°	0	0	0	0…−90	0…90	0
Distribution		-	homogeneous					heterog.

* The symbols refer to the Euler angles in the coordinate alignment process and the superscripts refer to the plane in which the rotation takes place.

## Data Availability

Not applicable.

## Appendix A

In this section the results of study 2.3 are shown in Figure A1, Figure A2, Figure A3 and Figure A4.
